# Information Bottleneck: Theory and Applications in Deep Learning

**DOI:** 10.3390/e22121408

**Published:** 2020-12-14

**Authors:** Bernhard C. Geiger, Gernot Kubin

**Affiliations:** 1 Know-Center GmbH, Inffeldgasse 13/6, 8010 Graz, Austria; 2 Signal Processing and Speech Communication Laboratory, Graz University of Technology, Inffeldgasse 16c, 8010 Graz, Austria; g.kubin@ieee.org

**Keywords:** information bottleneck, deep learning, neural networks

The information bottleneck (IB) framework, proposed in [1], describes the problem of representing an observation *X* in a lossy manner, such that its representation *T* is informative of a relevance variable *Y*. Mathematically, the IB problem aims to find a lossy compression scheme described by a conditional distribution $P_{T|X}$ that is a minimizer of the following functional:

(1) $$\min_{P_{T|X}}\left(I(X;T)-\beta I(Y;T)\right)$$

where the minimization is performed over a well-defined feasible set.

The IB framework has received significant attention in information theory and machine learning; cf. [2,3]. Recently, the IB framework has also gained popularity in the analysis and design of neural networks (NNs): The framework has been proposed to investigate the stochastic optimization of NN parameters with information–theoretic quantities, e.g., [4,5], and the IB functional was used as a cost function for NN training [6,7].

Based on this increased attention, this Special Issue aims to investigate the properties of the IB functional in this new context and to propose learning mechanisms inspired by the IB framework. More specifically, we invited authors to submit manuscripts that provide novel insight into the properties of the IB functional that apply the IB principle for training deep, i.e., multi-layer machine learning structures such as NNs and that investigate the learning behavior of NNs using the IB framework. To cover the breadth of the current literature, we also solicited manuscripts that discuss frameworks inspired by the IB principle, but that depart from them in a well-motivated manner.

In the remainder of this Editorial, we provide a brief summary of the papers in this Special Issue, in order of their appearance.

- Kunze et al. show that maximizing the evidence lower bound with a factorized Gaussian approximate posterior effectively limits mutual information between the available data and the learned parameters [8]. The effect of this tunable “model capacity” is validated in supervised and unsupervised settings, illustrating intuitive connections with overfitting, the NN architecture, and the dataset size;
- Wu et al. investigate the learnability within the IB framework. They show that if the parameter β in (1) falls below a certain threshold $\beta_{0}$, then a trivial representation *T* that is independent of *X* and *Y* minimizes the IB functional [9]. This threshold depends on the joint distribution of *X* and *Y*, and the authors propose an algorithm to estimate $\beta_{0}$ for a given dataset;
- Ngyuen and Choi argue that every layer in a feedforward NN should be optimized w.r.t. the IB functional (1) separately, with the parameter β adapted to the layer index [10]. Proposing a cost function for this multiobjective optimization problem, a computable variational bound, and a greedy optimization procedure, they achieve superior accuracy and adversarial robustness in stochastic binary NNs;
- Kolchinsky et al. propose an NN-based implementation of the IB problem, i.e., the compression scheme $P_{T|X}$ and conditional distribution $P_{Y|T}$ are parameterized by NNs [11]. Acknowledging the issues in [12], these NNs are trained to minimize an upper bound on ${\left(I\left(X;T\right)\right)}^{2}-\beta I\left(Y;T\right)$, combining variational and non-parametric approaches for bounding. Their experiments yield a better trade-off between I(X;T) and I(Y;T) and more meaningful latent representations in the bottleneck layer than a corresponding reformulation of [6];
- Tegmark and Wu investigate binary classification from real-valued observations [13]. They show that the observations can be compressed to a discrete representation *T* parameterized by β in such a way that the Pareto frontier of (1) is swept, essentially characterizing the binary classification problem. The authors further show that the corner points of this Pareto frontier, corresponding to a maximization of I(Y;T) for a given alphabet size of *T*, can be computed without multiobjective optimization;
- Rodríguez Gálvez et al. discuss the scenario in which the target *Y* is a deterministic function of *X* in [14]. In this case, it is known that sweeping the paramter β in (1) is not sufficient to sweep the Pareto frontier of optimal (I(X;T),I(Y;T)) pairs [12]. The authors show that this shortcoming can be removed by optimizing $u\left(I\left(X;T\right)\right)-\beta_{u}I\left(Y;T\right)$ instead, where *u* is a strictly convex function. Furthermore, the authors demonstrate that the particular choice of the strictly convex function *u* helps to obtain a desired value of I(X;T) over a wide range of parameters $\beta_{u}$;
- Franzese and Visintin propose using the IB functional as a cost function to train ensembles of decision trees for classification [15]. The authors show that these ensembles perform similarly to bagged trees, while they outperform the naive Bayes and *k*-nearest neighbor classifiers;
- Jónsson et al. [16] investigate the learning behavior of a high-dimensional VGG-16 convolutional NN in the information plane. Using MINE [17] to estimate I(X;T) and I(Y;T) throughout training, the authors observed a separate compression phase, during which the estimate of I(X;T) decreases, thus aligning with [4]. The authors further show that regularizing NN training via an MINE-based estimate of the compression term I(X;T) yields improved classification performance;
- Voloshynovskiy et al. propose an IB-based framework for semi-supervised classification, considering variational bounds both with learned and hand-crafted marginal distributions and achieving competitive performance [18]. A close investigation of their cost function yields improved insight into previously proposed approaches to semi-supervised classification;
- Fischer formulates the principle of minimum necessary information and derives from it the conditional entropy bottleneck functional [19]. This functional is mathematically equivalent to the IB functional, but uses the chain rule of mutual information to replace I(X;T) in (1) by I(X;T|Y). This results in different variational bounds, which are shown to yield better classification accuracy, improved robustness to adversarial examples, and stronger out-of-distribution detection than deterministic models or models based on variational approximations of (1), cf. [6];
- Fischer and Alemi provide additional empirical evidence for the claims in [19]. Specifically, they show that optimizing the proposed variational bounds leads to improved robustness against targeted and untargeted projected gradient descent attacks and to common corruptions (cf. [20]) of the ImageNet data [21]. Furthermore, the authors indicate that the conditional entropy bottleneck functional yields improved calibration for both clean and corrupted test data;
- Geiger and Fischer investigate the variational bounds proposed in [6,19]. While the underlying IB and conditional entropy bottleneck functionals are equivalent, the authors show that the variational bounds are not; these bounds are generally unordered, but an ordering can be enforced by restricting the feasible sets appropriately [22]. Their analysis is valid for general optimization and does not rely on the assumption that the variational bounds are implemented using NNs.

We thank all the authors for their excellent contributions and timely submission of their works. We are looking forward to many future developments that will build on the current bounty of insightful results and that will make machine learning better explainable.
